# Long-Term Effects of Bariatric Surgery on Gut Microbiota Composition and Faecal Metabolome Related to Obesity Remission

**DOI:** 10.3390/nu13082519

**Published:** 2021-07-23

**Authors:** María Juárez-Fernández, Sara Román-Sagüillo, David Porras, María Victoria García-Mediavilla, Pedro Linares, María Dolores Ballesteros-Pomar, Ana Urioste-Fondo, Begoña Álvarez-Cuenllas, Javier González-Gallego, Sonia Sánchez-Campos, Francisco Jorquera, Esther Nistal

**Affiliations:** 1 Instituto Universitario de Biomedicina (IBIOMED), Universidad de León, 24007 León, Spain; mjuarf@unileon.es (M.J.-F.); sroms@unileon.es (S.R.-S.); dpors@unileon.es (D.P.); mvgarm@unileon.es (M.V.G.-M.); jgonga@unileon.es (J.G.-G.); ssanc@unileon.es (S.S.-C.); fjorqueraplaza@gmail.com (F.J.); 2 Centro de Investigación Biomédica en Red de Enfermedades Hepáticas y Digestivas (CIBERehd), Instituto de Salud Carlos III, 28029 Madrid, Spain; 3 Servicio de Aparato Digestivo, Complejo Asistencial Universitario de León, 24071 León, Spain; plinares68@gmail.com (P.L.); bealvcue@gmail.com (B.Á.-C.); 4 Departamento de Endocrinología y Nutrición, Complejo Asistencial Universitario de León, 24071 León, Spain; mdballesteros@telefonica.net (M.D.B.-P.); anaurifon@gmail.com (A.U.-F.)

**Keywords:** bariatric surgery, gut microbiota, metabolomics, metagenomics, obesity, short-chain fatty acids

## Abstract

Obesity is one of the main worldwide public health concerns whose clinical management demands new therapeutic approaches. Bariatric surgery is the most efficient treatment when other therapies have previously failed. Due to the role of gut microbiota in obesity development, the knowledge of the link between bariatric surgery and gut microbiota could elucidate new mechanistic approaches. This study aims to evaluate the long-term effects of bariatric surgery in the faecal metagenome and metabolome of patients with severe obesity. Faecal and blood samples were collected before and four years after the intervention from patients with severe obesity. Biochemical, metagenomic and metabolomic analyses were performed and faecal short-chain fatty acids were measured. Bariatric surgery improved the obesity-related status of patients and significantly reshaped gut microbiota composition. Moreover, this procedure was associated with a specific metabolome profile characterized by a reduction in energetic and amino acid metabolism. Acetate, butyrate and propionate showed a significant reduction with bariatric surgery. Finally, correlation analysis suggested the existence of a long-term compositional and functional gut microbiota profile associated with the intervention. In conclusion, bariatric surgery triggered long-lasting effects on gut microbiota composition and faecal metabolome that could be associated with the remission of obesity.

## 1. Introduction

Obesity is one of the main public concerns worldwide, linked to increased rates of morbidity and mortality besides high resources demanding for public health systems [1]. Clinical management of overweight and obesity is traditionally based on lifestyle interventions due to the limited availability and efficacy of pharmacological therapies [2]. Bariatric surgery is considered the gold standard treatment when nonsurgical alternatives have failed, with great performance in remedying pathology in the short and in the long term [3,4]. Benefits of bariatric surgery exceed simple weight loss, counteracting other features of the metabolic dysfunction associated with obesity, including type 2 diabetes mellitus or hypertension [5].

The preponderant role of intestinal microbiota in the development of obesity is accepted in the current knowledge of the disease [6]. Under this perspective, the capacity of bariatric surgery to reshape gut microbiota as one of the mechanisms underlying its therapeutic success has been proposed, and several findings have been reported in this sense. For example, a greater microbial gene richness and bacterial diversity, features associated to a healthy microbiota, have been observed after bariatric surgery, although great interstudy and interindividual variability regarding changes in specific microbial taxa have been argued [7]. Moreover, the causal role of bariatric surgery-mediated changes in gut microbiota composition in its antiobesity effect has been demonstrated by reduced fat mass gain in germ-free mice colonized with faecal microbiota from operated donors [8].

One of the main contributions of the gut microbiota to the instauration of obesity resides in energy extraction from dietary nutrients. The microbiota of individuals with obesity is believed to have increased capacity for energy harvest [9], thus providing larger amounts of energetic metabolites such as short chain fatty acids (SCFAs) from plant polysaccharides [10]. Additionally, microbial metabolism of dietary nutrients may produce potential harmful substances related to the instauration of the low-grade inflammatory state of obesity [11]. Weight loss by means of dietary interventions modify gut microbiota, counteracting the extensively reported elevated Firmicutes/Bacteroidetes ratio in obesity and increasing beneficial Verrucomicrobia phylum [12], but also promoting functional changes in the microbiota and altering its derived metabolites [13,14]. Likewise, bariatric surgery could drive a shift of the metabolic capacity of the gut microbiota towards a lean-like phenotype, both from a composition and from a functional point of view, related to the good outcomes of the procedure.

Here we report the results of a longitudinal long-term study with a multiomics approach to characterise the impact of bariatric surgery on gut microbiota and associated metabolome in patients with clinically severe obesity.

## 2. Materials and Methods

### 2.1. Study Population

Nine patients with severe obesity were included in the study to analyze their status before and four years after bariatric surgery. All patients were characterized in detail from a demographic point of view (age, sex and race). The age range of the patients was between 20 and 60 years old, with two men and seven women, all of them Caucasians. They were also characterized from an anthropometric and biochemical point of view. Likewise, insulin resistance was determined using the homeostatic model assessment (HOMA-IR). None of the patients had been treated with antibiotics for at least one month prior to sampling.

The surgical procedures were laparoscopic vertical sleeve gastrectomy (VSG) (*n* = 6), biliopancreatic diversion (BPD) (*n* = 2) and gastric bypass (*n* = 1), selected according to the protocols of the High-Risk Obesity Unit from Complejo Asistencial Universitario of León (CAULE). Patient evolution was monitored by the Endocrinology and Digestive Diseases Services of the CAULE, evaluating the changes derived from the surgical intervention in the previously indicated parameters. Moreover, after surgery, patients underwent nutritional education by a registered dietitian. The study was conducted according to the guidelines outlined in the Declaration of Helsinki, and all procedures involving human subjects were approved by the Ethics Committee of the Complejo Asistencial Universitario de León (CAULE) (protocol code 2135, 23 February 2021). All patients gave informed written consent before participating in the study.

### 2.2. Sample Collection

**Figure 1:**
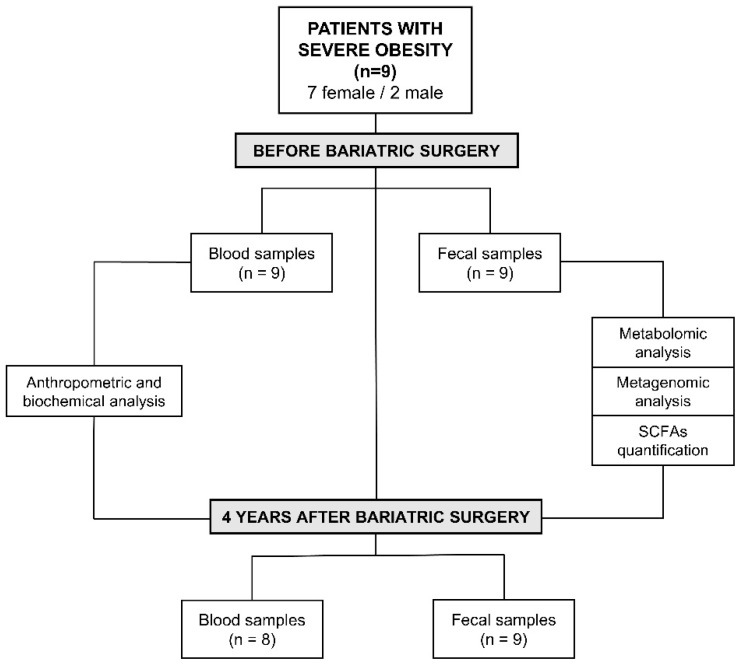
Experimental design and characteristics of the patients included in the study.

Blood samples of patients with severe obesity before and four years after bariatric surgery were collected to determine the above-mentioned biochemical parameters (Figure 1). After the intervention, a blood sample from one patient could not be collected, as illustrated in Figure 1.

Moreover, fresh stool samples were collected before and after surgical intervention from the nine patients included in the study (Figure 1). All samples were homogenized and aliquoted within three hours of defecation. The aliquots were stored at −80 °C until analysis.

### 2.3. Faecal Metagenomic Analysis

Total genomic DNA was isolated from faecal samples using the Qiagen Fast DNA Stool Mini Kit (Qiagen, Hilden, Germany) according to manufacturer instructions with some modifications previously described [15]. A mechanical lysis step was included based on an initial bead-beating step for 10 min, and lysis temperature was increased to 95 °C to facilitate DNA extraction from Gram positive bacteria. DNA concentration was determined by using a NanoDrop-1000 spectrophotometer (NanoDrop Technologies, Wilmington, DE, USA). DNA samples were stored at −20 °C until their analysis.

Amplification of the 16S rRNA V3-V4 hypervariable region was carried out by PCR as previously described [16]. Triplicate PCR assays for each sample were performed and purified with the Wizard^®^ Genomic DNA Purification kit (Promega, Madison, WI, USA) following the manufacturer’s instructions.

The resulting amplicons were cleaned, quantified and sequenced on an Illumina MiSeq platform. The Illumina bcl2fastq© program was used to demultiplex sequencing data. Fastp and FastQC v0.11.8 [17] tools were used to check for quality, adapter trimmed and filtered forward and reversing raw reads. Quantitative Insights into Microbial Ecology software (QIIME version 1.9.1 [18]) was used, including forward and reverse reads joining, chimera removal, data filtering and taxonomic annotation. To remove chimeric sequences from the reads, the Usearch6.1 algorithm was used [19]. These sequences were clustered into operational taxonomic units (OTUs) using UCLUST, with a similarity threshold of 0.97 [20], and were aligned using PyNast against the 16S reference database GreenGenes (version 13.8) [21] using default parameters [20].

### 2.4. Faecal Metabolomic Analysis

Sample analysis was carried out by MS-Omics as follows. The analysis was performed using a Thermo Scientific Vanquish LC coupled to a ThermoQ Exactive HF-MS. An electrospray ionization interface was used as the ionization source. Analysis was performed in negative and positive ionization modes. The UPLC was performed using a slightly modified version of the protocol described by Catalin et al. (UPLC/MS Monitoring of Water-Soluble Vitamin Bs in Cell Culture Media in Minutes, Water Application note 2011, 720004042en). Peak areas were extracted using Compound Discoverer 2.0.

### 2.5. SCFAs Identification and Quantification

Sample analysis was carried out by MS-Omics as follows. Samples were acidified using hydrochloric acid, and deuterium-labelled internal standards were added. All samples were analysed in a randomized order. Analysis was performed using a high polarity column (Zebron™ ZB-FFAP, GC Cap. Column 30 m × 0.25 mm × 0.25 μm) installed in a GC (7890B, Agilent, Wilmington, DE, USA) coupled with a quadropole detector (5977B, Agilent). The system was controlled by ChemStation (Agilent). Raw data was converted to netCDF format using Chemstation (Agilent), before the data was imported and processed in Matlab R2014b (Mathworks, Inc., Natick, MA, USA) using the PARADISe software earlier described [22].

### 2.6. Statistical Analysis

Student’s t test was employed to evaluate significant differences and *p* < 0.05 was considered significant for differences. From microbiota composition and metabolomic data, statistical significance was determined by the nonparametric Wilcoxon test with *p* < 0.05. A false discovery rate (FDR)-adjusted *p* < 0.05 was considered for significance. Statistical analyses were performed using SPSS 24.0 software for Windows (Chicago, IL, USA) and R software (R-project, Vienna, Austria).

## 3. Results

### 3.1. Effect of Bariatric Surgery on Biochemical and Anthropometric Clinical Characteristics of Patients with Severe Obesity

The characteristics and clinical data, as well as the evolution in body weight loss of the participants of the study before and after bariatric surgery are depicted in Table 1 and Table S1.

Bariatric technique enhanced biochemical and anthropometric parameters compared with measurements before surgery, showing statistical differences between patients with obesity before and after the intervention in the main parameters associated with obesity and metabolic syndrome. These included body weight and body mass index (BMI), body fat percentage, glucose, insulin and HOMA-IR, percentage of glycated haemoglobin (HbA1c), high density lipoprotein cholesterol (HDL), uric acid, aspartate transaminase (AST) levels and C reactive protein (CRP) concentration (*p* < 0.05) (Table 1). Moreover, the percentage of weight loss of our patients after four years of bariatric surgery was 32.13 ± 2.46%, with a body weight recovery of 6.10 ± 2.84%, compared with the point of maximum body weight loss, which was the second year after the intervention (Table S1).

### 3.2. Differences in Faecal Microbiota Composition Associated to Bariatric Surgery

**Figure 2:**
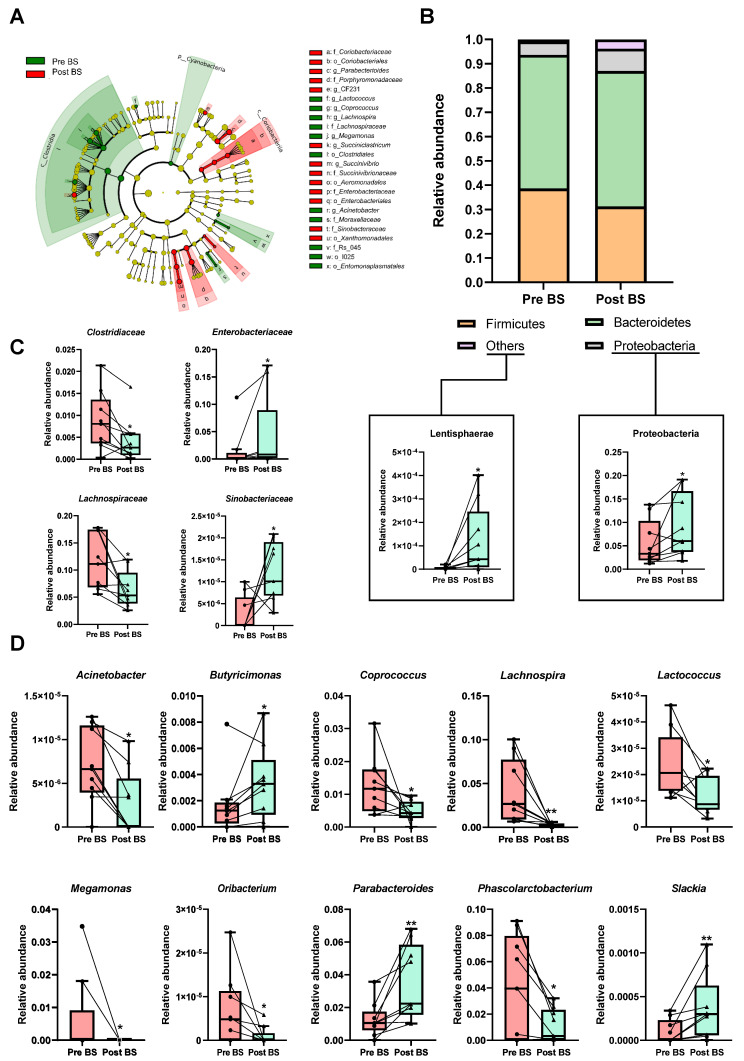
Effect of bariatric surgery on gut microbiota composition of patients with severe obesity. (**A**) Cladogram showing the main differences in gut microbiota composition before (Pre BS) and after (Post BS) bariatric surgery based on linear discriminant analysis effect size (LEfSe). Colour denotes taxa enriched in Pre BS samples (red) and Post BS samples (green). A cut-off value of LDA > 2 and a Wilcoxon *p* value < 0.05 were set up to identify discriminating taxa. The LEfSe results plot is provided in Figure S1. (**B**) Relative abundance of phyla in the patients with severe obesity Pre BS and Post BS. (**C**) Differences in the relative abundance at class level of the patients with severe obesity Pre BS and Post BS. (**D**) Differences in the relative abundance at genus level of the patients with severe obesity Pre BS and Post BS. Nonparametric Wilcoxon signed-rank test. * *p* < 0.05; ** *p* < 0.01 vs. Pre BS.

A cladogram was performed to show the main differences in gut microbiota composition before and after bariatric surgery based on the linear discriminant analysis (LDA) effect size (LEfSe) allowing us to identify which taxa were contributing to discrimination between groups (Figure 2A and Figure S1A). Eleven and 13 bacterial taxa enriched from patients with severe obesity before and after surgery, respectively, were identified. Enriched phylotypes from obese patients before intervention were predominantly from Cyanobacteria phyla and the Clostridia class, whereas most from obese patients after surgery were enriched principally from the Coriobacteriia class.

A principal coordinate analysis (PCoA) based on the Morisita Horn index was performed to analyse the influence of e bariatric surgery on bacterial community distribution at the OTU level. This analysis revealed a clear separation of the faecal microbiota according to the second axis based on the intervention, which accounted for 15.25% of the total variance (Figure S1B). Additionally, bariatric surgery tended to increase gut microbiota diversity as calculated by the Shannon index, though these differences were not significant (Figure S1C).

Bariatric surgery transformed gut microbiota composition at different taxonomic levels, highlighting a significant reduction in Firmicutes phylum in comparison to the same patients before the intervention, whereas, the relative abundance of Proteobacteria and Lentisphaerae phyla was significantly increased in response to bariatric procedure (Figure 2B). Changes were also detected at the family level, in that *Enterobacteriaceae* and *Sinobacteriaceae* were significantly increased in operated patients. On the contrary, *Clostridiaceae* and *Lachnospiraceae* families were reduced in post bariatric surgery patients (Figure 2C). The most remarkable differences were observed at the genus level. Interestingly, a higher detection of *Butyricimonas*, *Parabacteroides* and *Slackia* genera was observed after the intervention, while genera such as *Acinetobacter*, *Coprococcus*, *Lachnospira*, *Lactococcus*, *Megamonas*, *Oribacterium* and *Phascolarctobacterium*, which dominated in nonoperated obese patients, were significantly decrease after the surgical procedure (Figure 2D).

### 3.3. Effect of Bariatric Surgery on Faecal Metabolomic Profile of Patients with Severe Obesity

**Figure 3:**
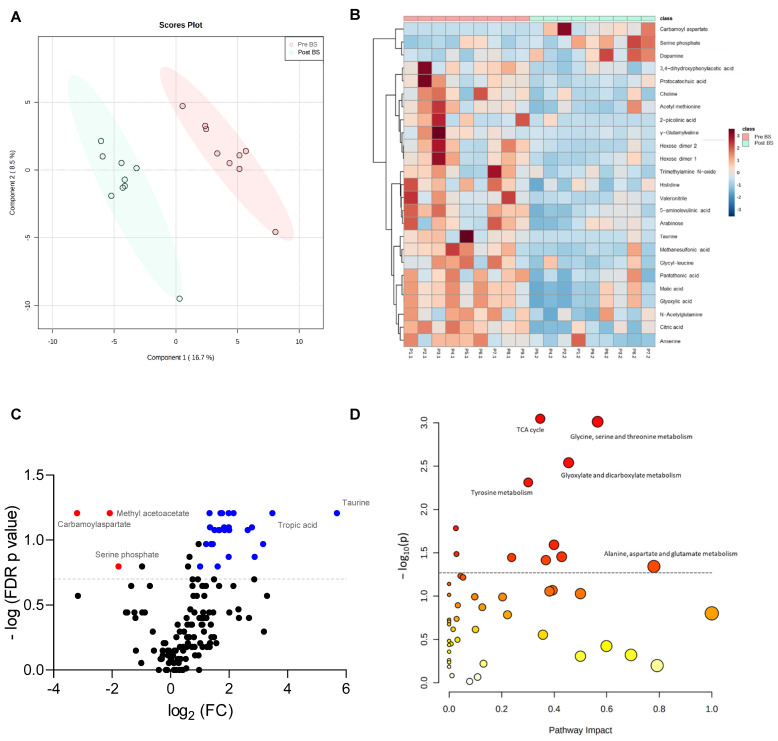
Effect of bariatric surgery on faecal metabolome of patients with severe obesity. (**A**) Partial Least-Squares Discriminant Analysis (PLS-DA) of faecal metabolites of patients with severe obesity before (Pre BS) and after (Post BS) bariatric surgery. (**B**) Heatmap showing normalized abundance of the top 25 discriminating faecal metabolites based on univariate t tests in the patients with severe obesity Pre BS and Post BS. Colour scale denotes higher (red) to lower (blue) abundance. Left side dendrogram represents correlations between metabolites based on Pearson’s correlation. (**C**) Volcano plot showing faecal metabolites with Wilcoxon signed-rank test *p* < 0.05 and fold-change (FC) >2 (positive FC in blue, negative FC change in red) due to bariatric surgery. (**D**) Pathway enrichment analysis showing the metabolic pathways significantly associated with surgery. The circle colour (from light yellow to red) is based on its *p*-value, and radius represents pathway impact value. Discontinue grey line denotes *p* value threshold of 0.05.

The Partial Least-Squares Discriminant Analysis (PLS-DA) of faecal metabolites showed a clear separation of the patients with severe obesity before and after bariatric surgery (accuracy 0.88889; R2 0.99074; Q2 0.5581), suggesting that the surgical intervention modified their faecal metabolome significantly (Figure 3A). The metabolites which allowed discrimination between both groups in the PLS-DA are included in Figure S2A as a Variable Importance in Projection (VIP) scores plot. Moreover, the individual representation of the normalized abundance of the top 25 discriminating faecal metabolites of each patient before and after the intervention in a heatmap showed a clear and different metabolomic profile depending on the bariatric surgery (Figure 3B).

To identify the metabolites that changed the most with bariatric surgery, a Volcano plot based on the Wilcoxon signed-rank test (*p* < 0.05, fold-change (FC) > 2) was performed, in which a total of 35 metabolites were identified to significantly change because of the intervention. As shown in Figure 3C, the abundance of methyl acetoacetate, carbamoyl aspartate and serine phosphate were significantly reduced with bariatric surgery, whereas other metabolites, such as taurine or tropic acid, were increased with the intervention. Bariatric surgery also diminished significantly the faecal concentration of 5-aminolevulinic acid, γ-glutamylvaline, acetamide, citric acid, glyoxylic acid, malic acid, maltotriose, N8-acetylspermidine, protocatechuic acid, trimethylamine-N-oxide (TMAO), valeronitrile and vanillin, among others (Figure S2B).

Furthermore, a pathway enrichment analysis based on the faecal metabolites detected in the patients of study was performed to identify the metabolic pathways which significantly changed due to bariatric surgery (Figure 3D). The tricarboxylic acid (TCA) cycle, glycine, serine and threonine metabolism, glyoxylate and dicarboxylate metabolism, tyrosine metabolism and the alanine, aspartate and glutamate metabolism (Figure 3D), were the pathways on which the bariatric surgery had the biggest impact.

### 3.4. SCFAs Faecal Profile before and after Bariatric Surgery of Patients with Severe Obesity

**Figure 4:**
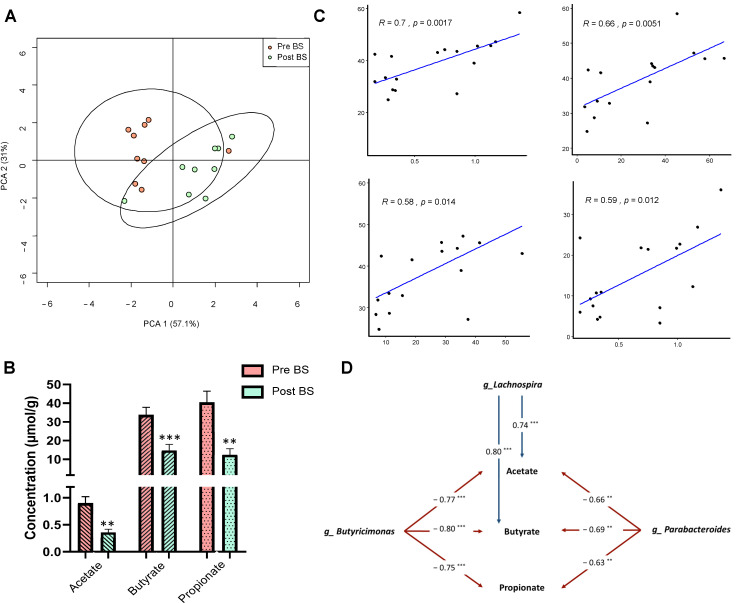
Faecal short-chain fatty acids profile before and after bariatric surgery in patients with severe obesity. (**A**) Principal coordinate analysis (PCA) showing the differences in the faecal short-chain fatty acids profile of patients before (Pre BS) and after (Post BS) bariatric surgery. (**B**) Differences in the faecal concentration (µmol/g of faeces) of the main short-chain fatty acids in patients with severe obesity Pre BS and Post BS. Nonparametric Wilcoxon signed-rank test. ** *p* < 0.01; *** *p* < 0.01 vs. Pre BS. (**C**) Pearson’s correlation coefficients and linear relationships of the main short-chain fatty acids (acetate, butyrate and propionate) and the biochemical and anthropometric parameters. (**D**) Associations of gut microbiota composition with the main short-chain fatty acids (acetate, butyrate and propionate). The values in arrows represent Spearman’s correlation coefficients of each pair of parameters. ** *p* < 0.01; *** *p* < 0.01. Detailed XY representation of each association is provided in Figure S3.

The SCFAs faecal profile of the patients was analysed by gas chromatography-mass spectrometry (GC-MS) to determine whether bariatric surgery had modified these molecules. As illustrated in Figure 4A, Principal Component Analysis (PCA) showed that the patients before bariatric surgery were grouped separately from patients after bariatric surgery according to the first axis, with accounted for 57.1% of the total variance. Among the SCFAs identified, the concentrations of acetate, butyrate and propionate were significantly reduced after bariatric surgery (Figure 4B).

### 3.5. Correlations between Biochemical and Anthropometric Parameters, Gut Microbiota Composition and Metabolomic Profile of Patients with Severe Obesity

Correlation analysis was performed to identify possible links between the benefits and improvements of bariatric surgery and the faecal SCFAs, metabolomic and metagenomic profiles.

Related to the faecal profile of SCFAs, Pearson’ correlation analysis between biochemical and anthropometric parameters that changed with bariatric surgery and the main SCFAs showed that acetate, butyrate and propionate significantly and positively correlated with body mass index (BMI), suggesting that the decrease in these metabolites is involved in the improvement of obesity. Moreover, insulin plasma levels also positively correlated with acetate concentration. (Figure 4C). Additionally, Spearman’ correlation analysis between SCFAs and gut microbiota composition at the genus level was performed. As shown in Figure 4D, the genera *Butyricimonas* and *Parabacteroides* negatively correlated with acetate, butyrate and propionate, whereas *Lachnospira* correlated positively. The detailed XY representation of each correlation is provided in Figure S3.

**Figure 5:**
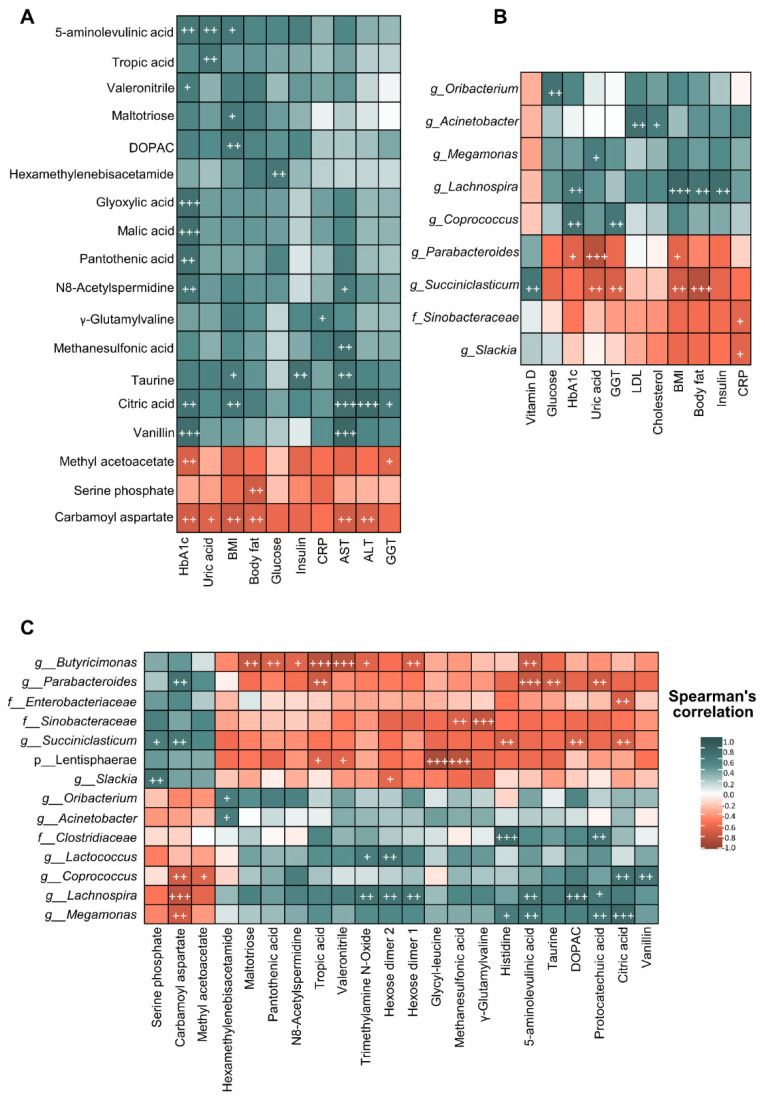
Correlation analysis between biochemical and anthropometric parameters, microbiota composition and the metabolomic profile of patients with severe obesity under bariatric surgery. (**A**) Heatmap of correlations between faecal metabolomic profile and faecal microbiota composition considering results of patients with severe obesity before and after bariatric surgery. (**B**) Heatmap of correlations between faecal metabolomic profile and biochemical and anthropometric parameters considering results of patients with severe obesity before and after bariatric surgery. (**C**) Heatmap of correlations between faecal microbiota composition and biochemical and anthropometric parameters considering results of patients with severe obesity before and after bariatric surgery. Each square represents the Spearman’s correlation coefficient (*p* < 0.01). Red and green cells designate positive and negative correlations, respectively. White crosses denote level of significance ^+^ *p* < 0.01, ^++^ *p* < 0.005, ^+++^ *p* < 0.001.

Moreover, correlation analysis of biochemical and anthropometric parameters, gut microbiota composition and faecal metabolites after bariatric surgery were performed using Spearman’s correlation (Figure 5). From a global point of view, bariatric surgery was associated with a specific metagenomic and metabolomic profile, which could be linked with the beneficial effects on biochemical and anthropometric parameters observed after the intervention. Spearman’s correlation between biochemical and anthropometric parameters and faecal metabolites highlighted that citric acid was positively correlated with HbA1c, BMI, AST and ALT, whereas carbamoyl aspartate showed an opposite pattern (Figure 5A). Furthermore, correlation analysis between biochemical and anthropometric parameters and gut microbiota taxa (Figure 5B) showed that the *Lachnospira* genus positively correlated with HbA1c, BMI, body fat and insulin while *Parabacteroides* and parameters like HbA1c, uric acid and BMI were negatively correlated. Finally, the heatmap performed between gut microbiota and faecal metabolites (Figure 5C) showed that *Lachnospira* positively correlated with different metabolites such as TMAO, carbamoyl aspartate, hexose dimers 1 and 2 and 5-aminolevulinic acid. In contrast, *Butyricimonas* correlated negatively with these metabolites, as well as with tropic acid and valeronitrile.

## 4. Discussion

In this study, we developed a longitudinal long-term analysis in which the faecal metabolome and metagenome of patients with severe obesity were determined before and after bariatric surgery to characterize the persistent effects of the intervention. First, our results showed that bariatric surgery had a profound effect on biochemical and anthropometric parameters, improving the health status of the patients four years after the intervention, as was previously described [23]. In this sense, bariatric surgery had a huge effect in body weight loss, ranging from 25–40% [24], findings that agree with the percentage of weight loss observed at the fourth year in our patients. Nevertheless, weight gain is a common complication after the intervention, with approximately one third of patients regaining excessive weight, which is defined as ≥25% of total lost weight [25]. In our study, a body weight regain of 6.10% at the fourth year was observed in the patients, a result which cannot be considered a remarkable regain.

Bariatric surgery not only has effects on clinical status, but also modulatory and reshaping effects on gut microbiota. Our results showed that bariatric surgery modified the gut microbiota profile of the patients with severe obesity, as previously reported [26,27,28,29]. At the phylum level, bariatric surgery reduced the abundance of Firmicutes phylum, whereas Proteobacteria showed an opposite pattern, reinforcing previous studies [30,31]. High concentrations of Firmicutes have been related with greater metabolic degradation of energy sources, which results in an increase of caloric absorption and, consequently, more weight gain. It should be noted that our patients were still considered at the border between overweight and obese at the end of the study according to their average BMI (≈30). In this sense, increased Firmicutes abundance has been consistently associated to obesity [32], so promoting a change towards a healthy profile (lower Firmicutes) may be a determinant of the beneficial effects of bariatric surgery. In contrast, the Proteobacteria phylum has been associated with a beneficial profile characterized by decreased systemic inflammation and improved glucose homeostasis [7], and correlated with weight loss [28]. Moreover, the increase in luminal pH due to the reduction in gastric volume following bariatric surgery may be one of the factors that allow the proliferation of this taxa [33].

Surgical intervention also modified gut microbiota composition at the class level, increasing the abundance of *Enterobacteriaceae*, which has been negatively correlated with cholesterol levels in humans [34,35] as well as positively correlated with weight loss in animal models [36], and whose increment has been previously reported after gastric bypass [37] and bilio-intestinal bypass [38]. Additionally, previous studies have postulated the reduction in the abundance of the *Clostridiaceae* family after bariatric surgery [36,38], which agrees with our results. On the contrary, the abundance of the *Lachnospiraceae* family members has been positively correlated with BMI, suggesting that the decreased abundance of this family in our study may be related to weight loss [39]. Surgical intervention also had a profound impact at the genus level. Thus, the decrease observed in the *Coprococcus* genus was previously found after different types of bariatric surgery [27,30], whereas the decreases observed in the *Lactococcus* genus were less consistent [27]. Nevertheless, high levels of *Lactococcus* have been associated with obesity and fasting plasma insulin [40]. Moreover, *Acinetobacter* overgrowth was present in patients under failed bariatric surgery [41], and in our study was positively correlated with LDL plasma concentration, so the significant reduction of this genus could indicate a more successful treatment. On the other hand, Faria et al. [42] reported that patients without weight regain after bariatric surgery presented an increased *Phascolarctobacterium* genus [42]. This finding does not agree with our results, because our patients showed a reduction in this genus, as well as a significant and persistent decrease in body weight four years after bariatric surgery. In contrast, bariatric surgery induced an increase in *Butyricimonas, Parabacteroides* and *Slackia* genera. *Parabacteroides* has been negatively correlated with serum insulin concentration after bariatric surgery [27] and with BMI in our study, whereas high levels of *Slackia* have been detected in patients after Roux-en-Y gastric bypass [35]. Furthermore, increased abundance of *Butyricimonas* has been associated with less food addiction after bariatric surgery [29]. Finally, *Lachnospira* decreased significantly in operated patients and was positively correlated with BMI, body fat and insulin levels, unlike a previous study in which *Lachnospira* was negatively correlated with BMI and fasting blood glucose [43]. Moreover, this genus correlated positively with TMAO and decreased with bariatric surgery, which is in accordance with its reported ability to produce this metabolite [44]. Furthermore, a recent study of the alterations on microbiota in childhood obesity showed that *Lachnospira* was significantly elevated in patients with obesity [45].

The faecal metabolome of the patients with severe obesity was also modified due to bariatric surgery, in accordance with previous findings [30,46], showing a different profile after four years of surgical intervention. In fact, methyl acetoacetate, carbamoyl aspartate and serine phosphate increased, whereas metabolites such as 5-aminolevulinic acid, choline, citric acid, malic acid, taurine, TMAO and tropic acid decreased in faeces, suggesting that long-term effects of surgical intervention affected this characteristic metabolomic profile. In this sense, previous studies described a reduction in choline [47,48], TMAO [48,49], taurine [50] or citric acid [49] with bariatric surgery, which agree with our results. Moreover, taurine was positively correlated with insulin and AST levels, whereas citric acid was correlated with the parameters AST, ALT and BMI. In this sense, taurine is an essential metabolite in bile acid formation, and high levels have been associated with obesity [51]. Related to the TMAO metabolite, this is derived from gut microbiota choline metabolism, whose high levels have been associated with organ damage and cardiovascular disease [49]. Although an increased concentration of this metabolite has been reported one year after bariatric surgery [48], our results showed that four years after intervention levels of TMAO were reduced, so this may be considered a long-term beneficial effect of bariatric surgery. Additionally, TMAO positively correlated with the presence of *Lactococcus* genus, which agrees with its role as a minor producer of this metabolite [44].

Moreover, exploring metabolomic pathways linked with metabolites that could be influenced by bariatric surgery, surgical intervention reduced the tricarboxylic acid cycle, glycine, serine and threonine metabolism, glyoxylate and dicarboxylate metabolism and tyrosine metabolism. These results suggest that branched-chain amino acids (BCAAs) and aromatic amino acids, as well as energetic metabolism, were downregulated with bariatric surgery, findings that have previously reported [49,52,53,54,55]. The reduction in BCAA levels as a consequence of bariatric surgery could be a normalization of the altered amino acid profile associated with obesity [49,52,53], which has been linked to an impairment of glucose homeostasis [48,49]. Furthermore, citric or malic acid were reduced after bariatric surgery, which was directly linked with the observed downregulation in energetic metabolism, and could be a consequence of the decreasing glucose input and increasing gluconeogenesis [53].

SCFAs are metabolites produced by gut microbiota fermentation from carbohydrates involved in various physiological process, such as lipogenesis *de novo* and gluconeogenesis or energy harvest [56]. The role of SCFAs in health and disease is still controversial and is not completely understood. Therefore, high levels of these metabolites have been related to cardiometabolic and hepatic health [56,57,58], but also have been associated with gut dysbiosis, gut permeability and excess adiposity [10,59]. Moreover, elevated concentrations of the main SCFAs have been linked with obesity development and with a major energy extraction from the diet [9,10,59]. In our study, the SCFA faecal profiles of the patients after bariatric surgery were modified, with significant decreases of acetate, butyrate and propionate concentrations, in accordance with previous reports [38,60] and with the reduction in the aforementioned energetic metabolic pathway. These SCFAs all positively correlated with BMI, reinforcing their reported role in obesity. Moreover, acetate, butyrate and propionate had a negative correlation with *Butyricimonas* and *Parabacteroides* genera, and a positive correlation with the *Lachnospira* genus. Although *Butyricimonas* is a butyrate producer, and this metabolite decreased in our study while the bacteria increased, there are many other bacteria involved in this process, which could explain these results. Additionally, the reduction in the *Lachnospira* genus agrees with the decrease in acetate, due to its role as a producer of this metabolite [61], and may represent a characteristic feature of weight loss because reduced abundance of SCFA producers (including *Lachnospira* genus) has also been reported in mildly obese patients under restrictive diets [39]. Furthermore, these changes could be attributed not only to a modification in gut microbiota but also to the reshaped metabolome after bariatric surgery. Altogether, correlation analysis showed a metagenomic and metabolomic profile related to bariatric surgery that could be involved in the beneficial effects observed on biochemical and anthropometric parameters.

This study presents some limitations. First, the difficulties in the collection of stool samples linked with the scarce number of patients. Second, male and female numbers were not equivalent. Although our results agree with current research, further studies with larger cohorts would be necessary to obtain more statistical power and to settle the relationship between gut microbiota and bariatric surgery. Another limitation is related to the surgical procedures, as more restrictive techniques such as sleeve gastrectomy could have different effects on gut microbiota than procedures with a malabsorptive component, such as BPD or gastric bypass. Because our sample was small, we did not consider these potential differences. Finally, although the patients underwent nutritional education by a registered dietitian, they were not under any specific diet, so the changes in gut microbiota composition could also be influenced by interindividual diet patterns. Related to this, collecting additional information about the lifestyle of the patients during this period (exercise, use of drugs, etc) would have been useful to guarantee that the specific changes observed in gut microbiota were due to bariatric surgery. In this sense, it has been previously reported that both diet and exercise have profound modulatory effects on gut microbiota composition [62] and are key factors in the success of bariatric surgery [63,64]. In fact, a higher protein diet has been shown to enhance satiety, body weight and fat loss in operated patients [64]. However, the consistency and homogeneity of our results across the individuals draws attention to a common cause of the modifications observed that should be attributed to bariatric surgery, independently of other source of interindividual discrepancies such as dietary patterns or exercise habits.

## 5. Conclusions

In conclusion, our findings point to bariatric surgery as a long-term modulator of gut microbiota, not only on its composition but also its functionality, promoting less efficient energy extraction from the diet as a possible mechanism linked to the persistent beneficial metabolic effects of a successful intervention. Nonetheless, more research and larger study populations are needed to determine the specific mechanisms by which the gut microbiota profile triggered by bariatric surgery is involved in the improvement of obesity.

## Figures and Tables

**Table 1:** Anthropometric and biochemical information before and after bariatric surgery of patients with severe obesity.

	Pre BS	Post BS	*p* Value
**Anthropometric parameters**			
Weight (kg)	124.98 ± 6.33	85.78 ± 5.81 ***	1 × 10^−6^
BMI (kg/m^2^)	45.46 ± 2.05	31.09 ± 1.81 ***	4 × 10^−6^
Body fat (%)	50.75 ± 1.03	36.54 ± 2.91 **	0.003
Waist circumference (cm)	130.63 ± 4.54	111.71 ± 7.05	0.059
Hip circumference (cm)	131.13 ± 8.83	102.17 ± 8.26 **	0.008
Systolic blood pressure (mm)	133.63 ± 3.23	118 ± 6.77	0.064
Diastolic blood pressure (mm)	87.38 ± 2.52	86.86 ± 5.82	0.966
**Biochemical parameters**			
Glucose (mg/dL)	91 ± 3.08	83 ± 2.49 **	0.009
Insulin (mUI/mL)	21.46 ± 3.12	7.08 ± 1.05 **	0.001
HOMA-IR	4.34 ± 0.59	1.45 ± 0.21 **	0.001
HbA1c (%)	5.76 ± 0.13	5.19 ± 0.15 **	0.009
Total cholesterol (mg/dL)	175.75 ± 6.39	161.88 ± 14.84	0.427
LDL-cholesterol (mg/dL)	105.5 ± 6.65	80.38 ± 11.35	0.120
HDL-cholesterol (mg/dL)	51.50 ± 3.01	66.5 ± 4.07 **	0.004
Triglycerides (mg/dL)	93.75 ± 13.67	73.38 ± 11.95	0.247
Uric acid (mg/dL)	5.68 ± 0.42	4.37 ± 0.34 *	0.033
Urea (mg/dL)	30.75 ± 2.70	31.75 ± 2.66	0.584
Creatinine (mg/dL)	0.72 ± 0.03	0.72 ± 0.07	0.933
AST (U/L)	19.38 ± 1.44	14.88 ± 1.11 *	0.013
ALT (U/L)	25.75 ± 3.60	20.75 ± 8.29	0.476
GGT (U/L)	31.38 ± 6.42	16.5 ± 4.57	0.059
Vitamin D (ng/mL)	27.25 ± 2.96	52.25 ± 10.58	0.063
CRP (mg/L)	19.63 ± 10.98	2.71 ± 1.37 *	0.017

Data are presented as mean ± SEM. Pre BS, pre bariatric surgery; Post BS, post bariatric surgery; BMI, body mass index; HOMA-IR, homeostatic model assessment for insulin resistance; LDL, low density lipoprotein; HDL, high density lipoprotein; AST, aspartate transaminase; ALT, alanine transaminase; GGT, gamma-glutamyl transferase; CRP, C reactive protein. * *p* < 0.05; ** *p* < 0.01; *** *p* < 0.001 vs. patients with obesity before bariatric surgery (Pre BS) by Student’s *t*-test.

## Data Availability

The data presented in this study are available on request from the corresponding author.

## Supplementary Materials

The following are available online at https://www.mdpi.com/article/10.3390/nu13082519/s1. Figure S1: Effect of bariatric surgery on gut microbiota composition of patients with severe obesity. Figure S2: Effect of bariatric surgery on faecal metabolome of patients with severe obesity. Figure S3: Associations of gut microbiota profile with faecal short-chain fatty acids concentration.
