# Supplementary material for: Long-Term Effects of Bariatric Surgery on Gut Microbiota Composition and Faecal Metabolome Related to Obesity Remission
Source: Nutrients. 2021 Jul 23;13(8):2519. doi: 10.3390/nu13082519 (PMC8397959; doi:10.3390/nu13082519)
Supplement: Supplementary file 1 [file nutrients-13-02519-s001.zip › nutrients-1275636-supplementary.pdf]

Supplementary materials

Long-term effects of bariatric surgery on gut microbiota composition and faecal metabolome related to obesity remission

M. Juárez-Fernández[1], S. Román-Sagüillo[1], D. Porras[1], M.V. García-Mediavilla[1,2], P. Linares[3], M.D. Ballesteros-Pomar[4], A. Urioste-Fondo[4], B. Álvarez-Cuenllas[3], J. González-Gallego[1,2], S. Sánchez-Campos[1,2], F. Jorquera[1,2,3], E. Nistal[1,2]

[1]Instituto Universitario de Biomedicina (IBIOMED), Universidad de León, León, Spain

[2]Centro de Investigación Biomédica en Red de Enfermedades Hepáticas y Digestivas (CIBERehd), Instituto de Salud Carlos III, Madrid, Spain

[3]Servicio de Aparato Digestivo, Complejo Asistencial Universitario de León, León, Spain

[4]Departamento de Endocrinología y Nutrición, Complejo Asistencial Universitario de León, León, Spain

SUPPLEMENTARY MATERIALS

Table S1 .... **2**

Figure S1 .... **3**

Figure S2 .... **4**

Figure S3 .... **5**

Table S1

Table S1. Evolution of body weight loss of the patients after bariatric surgery.

Body weight loss (%)			
**1st year**	**2nd year**	**3rd year**	**4th year**
-34.60 ± 2.44	-35.72 ± 2.77	-35.61 ± 3.10	-32.13 ± 2.46

Data are presented as mean ± SEM.

Figure S1

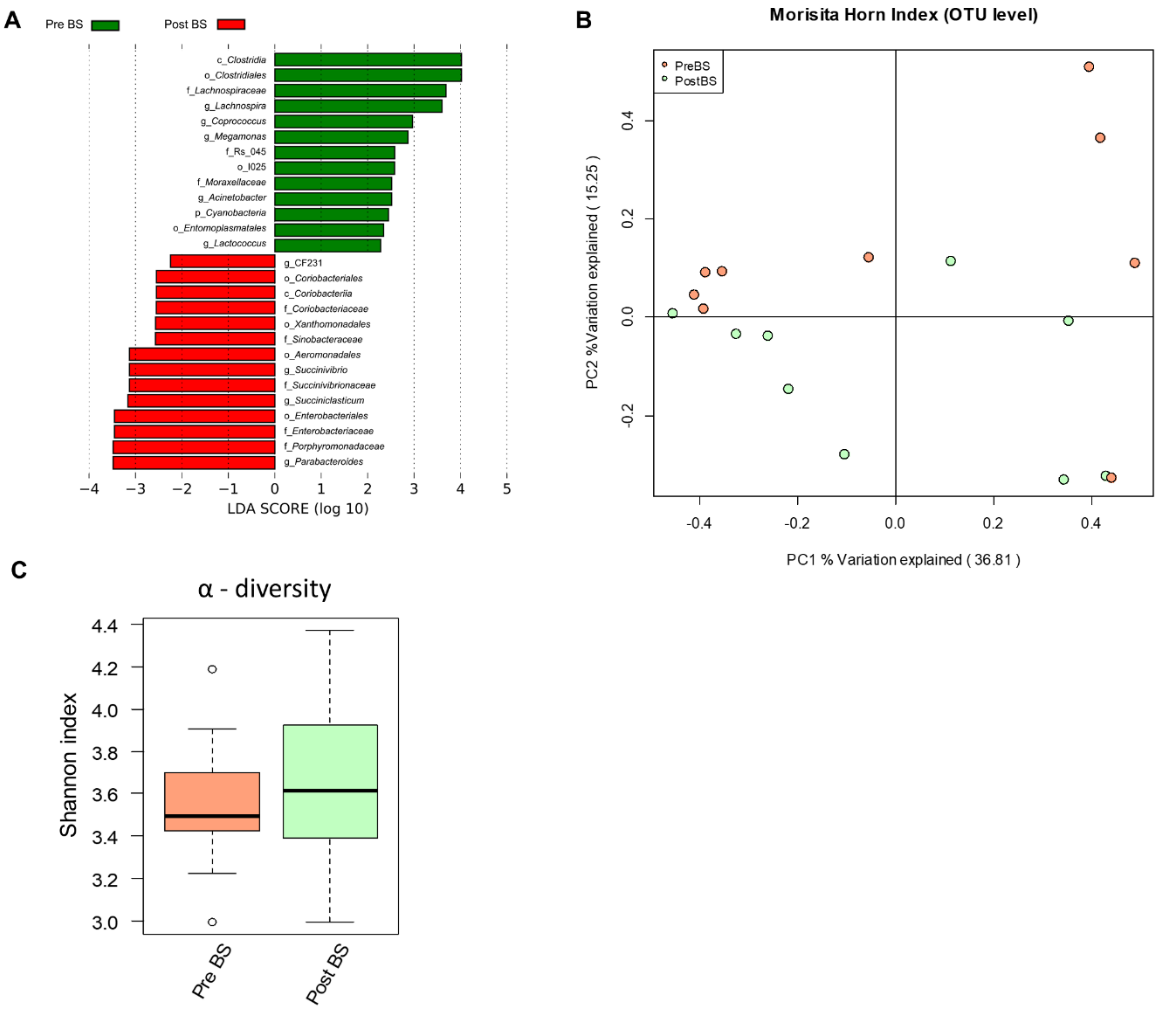

Figure S1. Effect of bariatric surgery on gut microbiota composition of patients with severe obesity. A. Linear discriminant analysis effect size (LefSe) showing differences between patients with severe obesity before (Pre BS) and after (Post BS) bariatric surgery. **B.** Principal coordinates analysis (PCoA) plot based on Morisita-Horn dissimilarity index at OTU level of patients with severe obesity Pre BS and Post BS. **C.** Differences in α-diversity measured by Shannon index of patients with severe obesity Pre BS and Post BS.

Figure S2

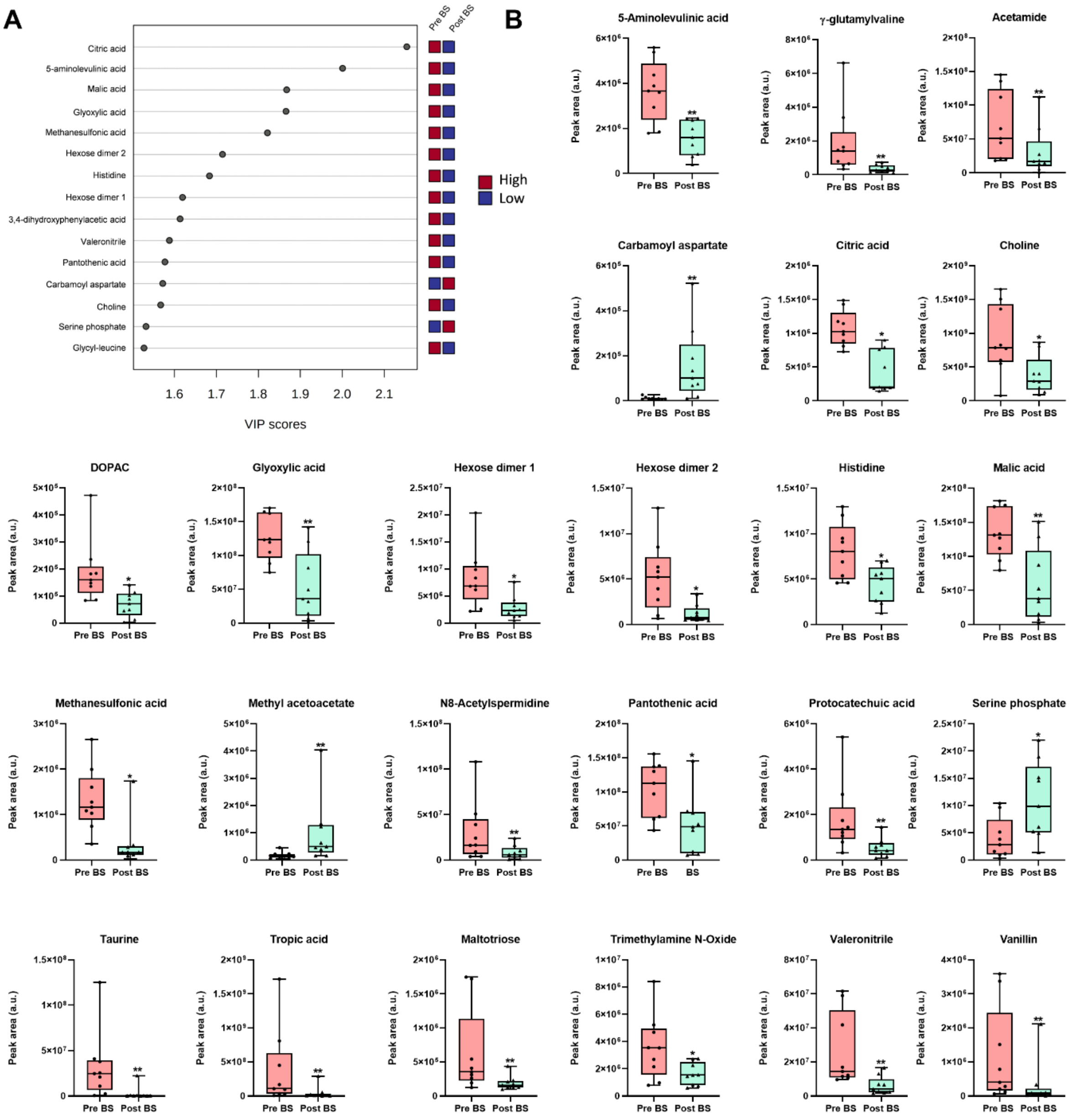

Figure S2. Effect of bariatric surgery on faecal metabolome of patients with severe obesity. A. Variable importance in projection (VIP) scores from Partial Least-Squares Discriminant Analysis (PLS-DA) of faecal metabolites of patients with severe obesity before (Pre BS) and after (Post BS) bariatric surgery. Red and blue boxes on the right indicate a high and low relative concentration, respectively, of the corresponding metabolite in each group. **B.** Boxplots showing differences in the peak area of different metabolites in patients with severe obesity Pre BS and Post BS. Non-parametric Wilcoxon signed-rank test. $^{*}p$<0.05; $^{**}p$<0.01 *vs.* Pre BS. $^{*}p$<0.05; $^{**}p$<0.01 *vs.* Pre BS.

Figure S3

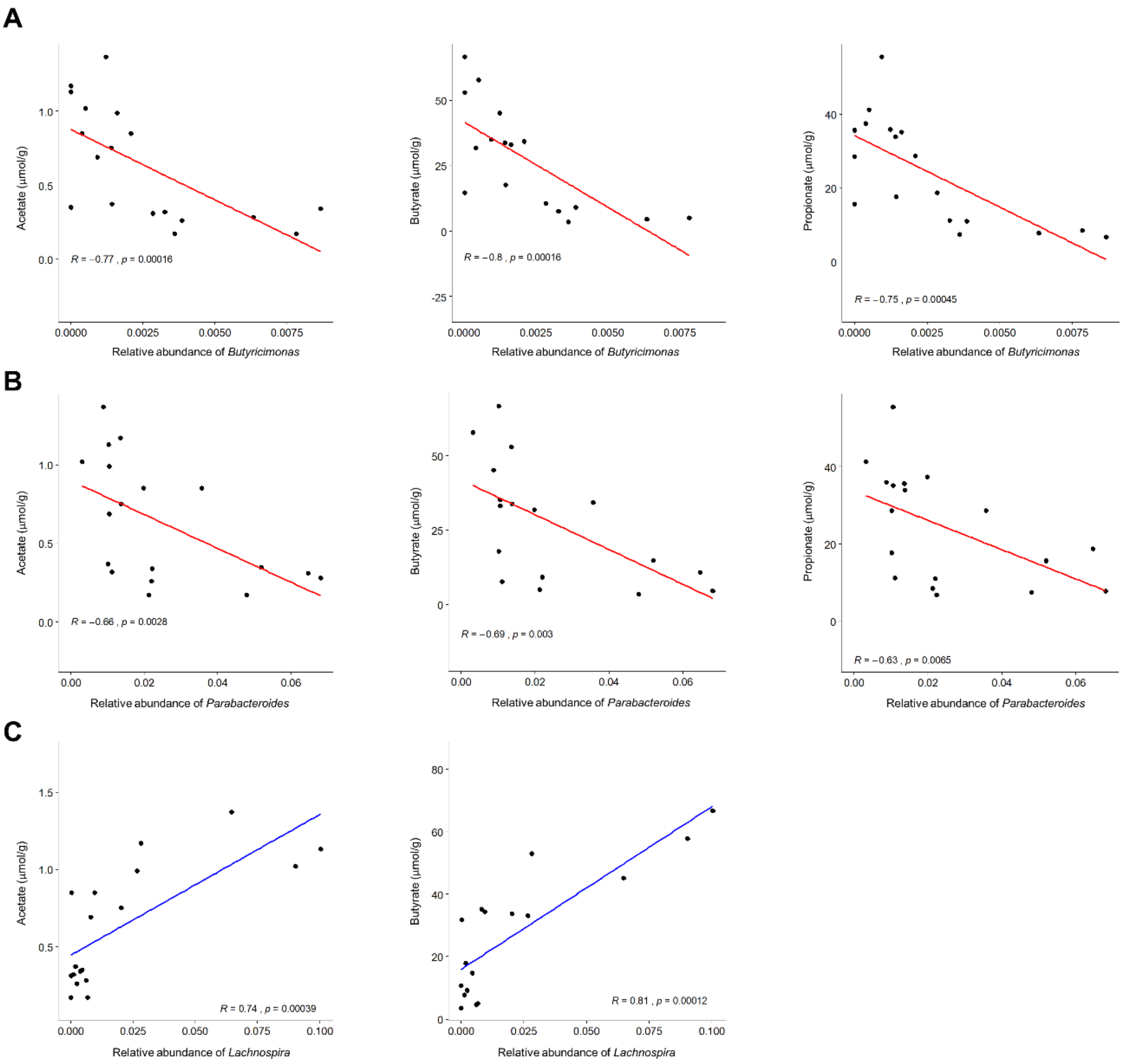

Figure S3. Associations of gut microbiota profile with faecal short-chain fatty acids concentration. Spearman correlation coefficients and linear relationships of **(A)** *Butyricimonas* relative abundance with acetate, butyrate and propionate concentrations; **(B)** *Parabacteroides* relative abundance with acetate, butyrate and propionate concentrations; **(C)** *Lachnospira* relative abundance with acetate, and butyrate. $^{*}p < 0.05$.
